# A Novel *SIL1* Variant (p.E342K) Associated with Marinesco–Sjögren Syndrome Impairs Protein Stability and Function

**DOI:** 10.3390/ijms262311310

**Published:** 2025-11-22

**Authors:** Anna Giulia Ruggieri, Nikolaos M. Marinakis, Laura Amodei, Francesca Potenza, Afrodite Kampouraki, Faidon-Nikolaos Tilemis, Laura Pietrangelo, Marianna Viele, Federica Di Marco, Piero Del Boccio, Federica Di Cintio, Nikoletta Selenti, Manthoula Valari, Luca Federici, Adriana Erica Miele, Michele Sallese, Periklis Makrythanasis

**Affiliations:** 1 Department of Innovative Technologies in Medicine and Dentistry, “G. d’Annunzio” University of Chieti-Pescara, 66100 Chieti, Italy; annagiulia.ruggieri@unich.it (A.G.R.); federica.dimarco@unich.it (F.D.M.);; 2 Center for Advanced Studies and Technology (CAST), “G. d’Annunzio” University of Chieti-Pescara, 66100 Chieti, Italy; p.delboccio@unich.it (P.D.B.);; 3 Laboratory of Medical Genetics, Medical School, St. Sophia’s Children’s Hospital, National and Kapodistrian University of Athens, 115 27 Athens, Greeceftilemis@med.uoa.gr (F.-N.T.); pmakryth@med.uoa.gr (P.M.); 4 Laboratory of Genetics, Department of Medicine, Democritus University of Thrace, 681 00 Alexandroupolis, Greece; 5 Department of Medicine and Aging Sciences, “G. d’Annunzio” University of Chieti-Pescara, 66100 Chieti, Italy; laura.pietrangelo@unich.it; 6 Department of Sciences, “G. d’Annunzio” University of Chieti-Pescara, 66100 Chieti, Italy; 7 Department of Medical, Oral and Biotechnological Sciences, “G. d’Annunzio” University of Chieti-Pescara, 66100 Chieti, Italy; 8 Department of Dermatology, Medical School, St. Sophia’s Children’s Hospital, National and Kapodistrian University of Athens, 115 27 Athens, Greece; 9 Department of Biochemical Sciences, Sapienza University of Rome, 00185 Rome, Italy; adriana.miele@univ-lyon1.fr; 10 Institute of Analytical Sciences, UMR 5280 ISA CNRS UCBL, Université Claude Bernard Lyon 1, 69100 Villeurbanne, France

**Keywords:** neurodegenerative disease, ataxia, myopathy, chaperones, proteomics, variant of unknown significance, VUS

## Abstract

Marinesco–Sjögren syndrome (MSS) is a rare autosomal recessive neuromuscular disorder marked by ataxia, muscle weakness, cataracts, and often intellectual and skeletal abnormalities. It is commonly caused by loss-of-function variants in the *SIL1* gene, which impair binding immunoglobulin protein (BiP) function, leading to protein misfolding and activation of the unfolded protein response. In a 2-year-old patient with typical MSS symptoms, we identified a previously unreported c.1024G>A (p.E342K) variant in *SIL1* via whole-exome sequencing. The pathogenicity of this Sil1 variant was supported by evidence of structural changes revealed through in silico predictions, circular dichroism, and native gel electrophoresis. Patient-derived fibroblasts exhibited reduced Sil1 protein levels, likely due to misfolding and degradation, which was partially rescued by proteasome inhibition. Proteomics revealed a profile similar to known MSS cases and a distinctive MSS transcriptional signature. Ultrastructural analysis confirmed typical MSS features, such as autophagic vacuoles and lipid droplets. Although the p.E342K phenotype appears milder than the reference pathogenic variant R111X, our findings support the reclassification of this novel variant as pathogenic, in accordance with the American College of Medical Genetics and Genomics/Association for Molecular Pathology (ACMG/AMP) 2015 guidelines and the refinements proposed by the Clinical Genome Resource Sequence Variant Interpretation (ClinGen SVI) recommendations. Furthermore, the overall evidence also provides important insights into the genotype–phenotype correlation and the underlying pathogenic mechanism of the p.E342K variant.

## 1. Introduction

Marinesco–Sjögren syndrome (MSS) is a rare autosomal recessive neuromuscular disease with early onset [1,2,3,4]. The main clinical manifestations, called clinical triad, that are also central to the diagnosis, include ataxia and cerebellar atrophy, myopathy with muscle weakness, and congenital or early-onset cataract, often bilateral. Unfortunately, this triad does not always manifest, while other clinical signs may be present that complicate the diagnosis and confuse it with other forms of spastic paraplegia [5]. Indeed, MSS patients may show many other issues, including mild to severe intellectual disability, hypergonadotropic hypogonadism, short stature, skeletal abnormalities, strabismus, and nystagmus [2,6,7]. Therefore, whole-exome sequencing (WES) is an important option to support diagnosis in these complex phenotypes [5]. However, this approach raises the problem of associating variants of unknown significance (VUS) with the observed phenotypes.

In more than 50% of cases, MSS patients carry loss-of-function variants in the *SIL1* gene [7,8,9]. The *SIL1* gene product acts as a nucleotide exchange factor (NEF) for the endoplasmic reticulum (ER) chaperone called BiP [10]. Loss of Sil1 impairs BiP-mediated protein folding, leading to accumulation of unfolded proteins in the ER and activation of the unfolded protein response (UPR) [11,12,13]. While the UPR aims to restore ER homeostasis, skeletal muscle and Purkinje neurons are unable to resolve the stress and undergo apoptosis, although the exact mechanism remains unclear [14,15,16].

As of March 2025, 376 variants in the *SIL1* gene have been reported, of which 29 are described as clearly pathogenic, 10 as probably pathogenic, 151 as probably benign, 35 as benign and 193 as of uncertain significance [17,18]. Even if pathogenic variants comprise nonsense, frameshift, nucleotide duplication, and disruption of splicing sites, they more frequently lead to the synthesis of truncated proteins that are likely to be degraded. Variants are often located in exons 6 to 9, which encode for armadillo repeats, a critical region for the interaction with BiP [19]. Interestingly, exon 10, another variant-prone area, is also involved in contact with BiP. As mentioned, significant heterogeneity in the clinical signs of MSS patients has been described, although a clear genotype–phenotype relationship has not been emphasised [20]. The disambiguation of *SIL1* VUS is further complicated by both intrinsic and extrinsic factors, notably the paucity of documented patients, which constrains genotype–phenotype correlations and prevents robust segregation studies. Moreover, the absence of standardised and validated functional assays that fulfil the American College of Medical Genetics and Genomics/Association for Molecular Pathology (ACMG/AMP) PS3 (Pathogenic Strong) and BS3 (Benign Strong) criteria limited reliable interpretation of *SIL1* variants [21].

Nevertheless, available in vitro assays play a crucial role in investigating the pathogenicity of novel *SIL1* variants and therefore valuable for the differential diagnosis with other overlapping genetic syndromes, like hereditary spastic paraparesis type 46, or variants in INPP5K [22]. Expression of pathogenic Sil1 mutants in heterologous systems, such as HEK293 or COS7 cells, undergoes aggregation, unlike wild type (WT) Sil1, which is evenly distributed within the ER [23,24,25]. Furthermore, cells overexpressing Sil1 WT slightly activate the ER stress response, whereas those overexpressing pathogenic Sil1 mutants strongly activate the UPR and can trigger a pathogenic phenotype that includes an enlargement of the ER and an increase in the number of autophagic vacuoles [24].

In this study we identified for the first time a *SIL1* variant (c.1024G>A corresponding to p.E342K) in a 2-year-old Greek female patient. In silico analysis suggested a pathogenic role for this VUS. Recombinant Sil1 p.E342K appeared rather unfolded on circular dichroism analysis, showed abnormal separation on native gel electrophoresis, and formed aggregates when expressed in COS7 cells, thus confirming the potential pathogenicity of this variant. Fibroblasts isolated from the patient carrying the p.E342K variant showed 10 times less Sil1 protein, which increases when the cells are treated with a proteasome inhibitor. Lysosomal vacuoles were also increased as shown by ultrastructural analysis. These fibroblasts showed a characteristic transcription signature consistent with that of a patient carrying a recognised pathological *SIL1* variant [26]. Furthermore, proteomic analysis showed an abnormal protein expression pattern similar to that we previously reported [27]. Overall, the collective evidence, evaluated according to the ACMG/AMP 2015 guidelines and the refinements proposed by the Clinical Genome Resource Sequence Variant Interpretation (ClinGen SVI) recommendation, supports the reclassification of the novel *SIL1* c.1024G>A (p.E342K) variant as pathogenic [21]. 

## 2. Results

### 2.1. Clinical and Diagnostic Findings in a Patient with Suspected MSS

The proband’s antenatal and birth history were uneventful. She was born at 38 weeks of gestation via caesarean section. Her birth parameters, while within normal limits, were on the lower end of the spectrum, with a birth length at the 3rd–15th percentile, weight at the 15th percentile, and head circumference at the 15th percentile. At 1 year of age, concerns about hypotonia and developmental delay led to the initiation of occupational therapy. At 2 years, the proband demonstrated significant speech delay, with no meaningful verbal communication, and exhibited motor delay, being unable to sit unsupported and walk independently, taking only two steps with assistance.

Clinical examinations revealed astigmatism, hypermetropia, and residual esotropia in the left eye on ophthalmological assessment. Audiological and cardiological evaluations were unremarkable. Brain magnetic resonance imaging showed cerebellar atrophy, while blood counts, liver function tests, and renal function tests were normal.

At 3 years, a developmental assessment confirmed global developmental delay. The proband had significant difficulties with understanding and following commands, object recognition, and communication. She was non-verbal, unable to grasp objects appropriately, did not sit independently, and was unable to walk without support. Cognitive skills were at the developmental age of 15 months, speech perception and expression at less than 6 months, and fine motor skills at the developmental age of 12 months. There were concerns regarding potential epilepsy due to abnormal movements during sleep, although no formal diagnosis was made. Ophthalmological findings at 3 years showed only astigmatism. Electromyography suggested myopathic changes in the dynamic motor units of the quadriceps, while electroneurography was normal.

At her most recent follow-up at 4 years of age, the proband had shown no significant improvement in motor function. She was, however, able to pronounce 15–20 words and continued to receive both occupational and speech therapy. Her growth remained in the lower percentiles, with weight and head circumference between the 3rd and 15th percentiles, while her height had slightly improved, now at the 15th percentile. A hypercaloric diet with ground food and a caloric supplement was considered the most suitable nutritional option. A detailed family history revealed that the maternal half-sister has a history of epilepsy, psychological disorders, and behaviours consistent with autism spectrum disorder. She attended a special education school but had no motor disabilities or growth abnormalities.

**Figure 1:**
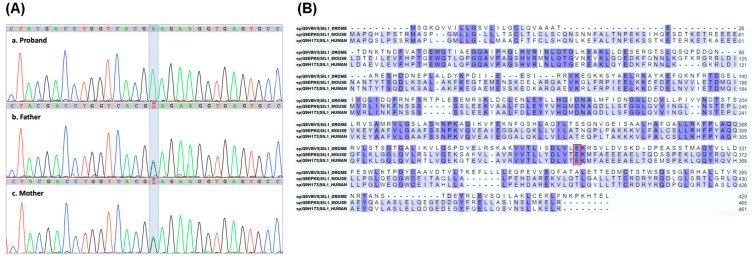
**Identification of a new c.1024G>A *SIL1* gene variant.** (**A**) Sanger sequencing electropherograms showing the *SIL1* gene region containing the c.1024G>A variant. The variant base in the proband and her parents is highlighted. (**B**) Alignment of Sil1 proteins from Drosophila, mouse, and human, highlighting the evolutionary conservation of the altered amino acid (p.E342K) encoded by the *SIL1* gene variant, boxed in red. Residues conserved across all three species are highlighted in dark violet, whereas those conserved in only two species are in light violet.

Applying WES, variant analysis, and filtering criteria (phenotype, population frequency, variant type, in silico prediction, etc.) as described in the methods, a new homozygous missense variant ΝΜ_022464.5:c.1024G>A, in the *SIL1* gene, was identified. This *SIL1* variant was confirmed through targeted Sanger sequencing of the proband and her parents (Figure 1A). According to ACMG criteria this variant was classified primarily as a VUS (PM2, PP3), noting that further investigation is needed for final evaluation. Targeted family segregation revealed that both parents were carriers for this variant in the *SIL1* gene.

### 2.2. In Silico Analysis Indicates That the c.1024G>A Variant Modifies the Structure of Sil1, Thereby Contributing to Its Pathogenic Role

To further investigate the possible clinical consequences of the c.1024G>A variant, we performed a search in the ClinVar database [28,29]. Unfortunately, this variant was confirmed as novel and therefore not reported in this database. In order to understand the possible effects of such a variant, we evaluated its potential pathogenicity using algorithms that predict the possible impact of amino acid substitutions on protein structure and function. According to the Polymorphism Phenotyping v2 [30,31,32], c.1024G>A is probably damaging, with a score of 1 on a scale ranging from 0 to 1. Mutation Taster [33,34] also predicted that the c.1024G>A variant is disease-causing with a probability of 0.99 (scale 0 to 1).

**Figure 2:**
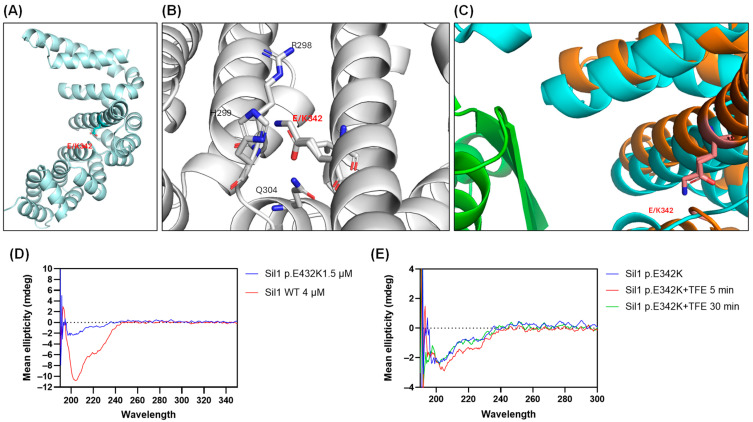
**Three-dimensional analysis of the Sil1 p.E342K variant by AlphaFold modelling and circular dichroism (CD) spectroscopy.** (**A**) AlphaFold modelling of the Sil1 p.E342K variant, with the position of the altered amino acid marked. The backbone atoms of Glu342 are shown in light cyan, and its side-chain functional group is rendered in red. (**B**) Zoomed-in view of the region surrounding amino acid 342 of Sil1, illustrating the position and electrostatic interactions of the mutant residue. A subset of amino acids residues surrounding the p.E342K variant is numbered. Backbone atoms appear in grey, with side-chain functional groups highlighted in red for acidic residues and in blue for basic residues. (**C**) Superposition of the human Sil1 model (shown in orange) with the complex between yeast Sil1 (cyan) and BiP (green). (**D**) CD plot of recombinant Sil1 WT and Sil1 p.E342K variant showing their structural differences. (**E**) Effect of trifluoroethanol (TFE) on the structure of the Sil1 p.E342K variant at 5 and 30 min, as analysed by CD.

The c.1024G>A *SIL1* variant causes the substitution of the conserved glutamic acid (Glu) into a lysine (Lys) at position 342 of the protein (p.E342K) (Figure 1B). This modification could have an impact on protein structure and function because it replaces a negatively charged amino acid with a positively charged one within the armadillo repeats helical domains. To investigate this possibility, we generated a model of the structure of human Sil1 through Alphafold [35]. Figure 2A shows a ribbon representation of the C-terminal folded domain (residues 179 to 461) showing the position of p.E342K approximately in the centre of a helix located in the middle of the concave surface implicated in BiP recognition. A (zoom) view of the area around residue 342 (Figure 2B) highlights the position and electrostatic interaction of the mutant residue. Its carboxylic side chain is at H-bond distance with both the peptide nitrogen of histidine 299 (His299) (2.91 Å) and the side chain of glutamine 304 (Gln304) (2.96 Å). His299 and arginine 298 (Arg298) side chains also contribute to establishing a positively charged pocket where the Glu342 side chain dwells. The Glu to Lys variant is obviously disturbing this network of interactions because of multiple electrostatic repulsions, and it is likely locally destabilises the whole area. To quantify the impact of the p.E342K variant on protein stability, we utilised the web server “Impact of Non-Synonymous Variations on Protein Stability-MultiDimension” [36,37]. The calculated change in protein stability (ΔΔG) was −1.062 kcal/mol, indicating a destabilising effect of the mutation.

Figure 2C shows a superposition of the above-mentioned human Sil1 model (shown in orange) with the complex between yeast Sil1 (cyan) and BiP (green) (DOI:10.2210/pdb3QML/pdb) [38]. Even though not directly interacting with BiP, Glu342 belongs to a helix that, as also highlighted above, contributes to forming a Sil1 profound cleft, where a two-stranded beta-sheet of BiP intercalates. It is therefore plausible that a destabilisation of this portion of Sil1, albeit only local, may result in compromising or weakening the interaction with BiP.

### 2.3. The Amount of Alpha-Helical Structures Is Reduced in the p.E342K Variant

Sil1 p.E342K and Sil1 WT, without their N-terminal signal peptides, were expressed in *E. coli* with a glutathione S-transferase (GST) tag at the N-terminus and purified by affinity chromatography as described in the Methods.

From the beginning, the Sil1 variant seemed less soluble than the WT protein. Following final purification and enzymatic removal of the GST tag, it proved to be highly unstable. For example, despite various attempts—including buffer adjustments, high salt concentrations, and the addition of glycerol—it was impossible to concentrate the recombinant p.E342K variant beyond 1.5 μM. In fact, when the protein was concentrated above this threshold, it precipitated, whereas the recombinant Sil1 WT protein could be concentrated up to 60 μM without any issues.

The 3D organisation of both recombinant p.E342K and WT proteins by measuring the circular dichroism (CD) spectra showed a folded structure. However, the CD profile of Sil WT was consistent with mixed alpha-beta folding, while that of p.E342K showed a loss of alpha-helix structure (Figure 2D). The CD spectrum of p.E342K was more reminiscent of a coiled-coil fold, which could explain its extreme instability (Figure 2D). Given the possible absence of alpha helices in the p.E342K variant, we wanted to test whether the WT conformation could be restored by adding trifluoroethanol (TFE), a stabiliser of helical structures [39]. Indeed, TFE transiently produced the appearance of a small negative peak around 222 nm (as if some helical component was induced to form), nevertheless it disappeared after 30 min of incubation (Figure 2E).

To sum up, the replacement of negatively charged glutamic acid at position 342 with a positively charged lysine strongly destabilises the Sil1 protein, resulting in a mutant with possible loss of function.

### 2.4. The p.E342K Variant Is Poorly Soluble and Forms Intracellular Aggregates When Expressed in COS7 Cells

Previous studies indicated that pathological Sil1 variants migrate abnormally in the native electrophoresis gel and form aggregates when expressed exogenously in COS7 or HEK293 cells [23,24,25]. We therefore decided to perform similar experiments with the aim of understanding whether the p.E342K variant behaves as a pathological or physiological protein.

**Figure 3:**
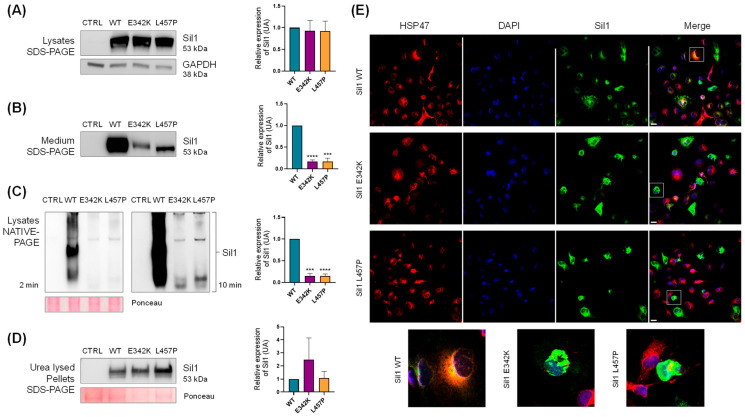
**Solubility analysis of the Sil1 p.E342K variant.** COS7 cells were transiently transfected with *SIL1* WT, p.E342K, and p.L457P mutants. Transfected proteins in the image are labelled with the abbreviations WT, E342K, and L457P, respectively. (**A**) Western blot analysis of whole-cell lysates from mock (CTRL), WT, p.E342K, and p.L457P transfected cells showed comparable expression levels of all three constructs. Glyceraldehyde-3-phosphate dehydrogenase (GAPDH) expression was analysed as loading control. (**B**) Western blotting of conditioned media revealed a reduced release of the p.E342K and p.L457P mutants into the culture medium. (**C**) COS7 cells transfected as described in A were lysed in native buffer and analysed by Native-PAGE and Western blotting. The left and right panels show the Western blotting of CTRL, WT, p.E342K, and p.L457P exposed for 2 and 10 min, respectively. Ponceau red was used as loading control. (**D**) Western blotting was performed on the insoluble pellet remaining after the native lysis described in C. This pellet was subjected to urea-based lysis to perform the analysis. Ponceau red was used as loading control. (**A**–**D**) Quantification of Sil1 expression from the Western blot is shown in the graphs on the right. Protein expression relative to WT are shown, normalised to GAPDH or Ponceau Red as indicated. Unpaired t-test was used to compare Sil1 protein expression of p.E342K and p.L457P mutants to WT. *** *p* < 0.001 and **** *p* < 0.0001. (**E**) Confocal IF analysis of COS7 cells transiently transfected with *SIL1* WT, p.E342K, and p.L457P mutants. The cells were fixed and stained for Sil1 (green), heat shock protein 47 (red) as an ER marker, and 4′,6-Diamidino-2-phenylindole dihydrochloride (DAPI) (blue) to visualise the nuclei. Merged images are also shown. Enlarged images of the cells are shown in the lower part of the figure to better appreciate the different intracellular distribution of Sil1 WT and mutants. Scale bar: 20 µm.

We first assessed whether the WT, p.E342K, and the pathogenic p.L457P mutant of Sil1 were expressed at comparable levels when transfected into COS7 cells. Western blotting on sodium dodecyl sulphate-sds of the COS7 cell lysates confirmed similar expression of the three Sil1 proteins (Figure 3A). If overexpressed, proteins that are normally resident in the ER lumen can overwhelm the ER retention mechanisms, leading to their release into the cell culture medium. In fact, Western blot analysis of the conditioned medium from COS7 cells transfected with the three *SIL1* cDNAs revealed the release of recombinant Sil1. However, both p.E342K and p.L457P variants were released into the culture medium in approximately 10-fold lower amounts than Sil1 WT (Figure 3B). This suggested possible solubility problems with both the p.E342K variant and the pathological mutant p.L457P.

In parallel experiments, COS7 cells were transfected with the constructs described above, lysed in native cell lysis buffer, separated by native gel electrophoresis, and recombinant Sil1 was detected by Western blotting. The amount of p.E342K and p.L457P variants was very low, about 10 times lower than that of Sil1 WT (Figure 3C).

To confirm that the mutant proteins were expressed similarly to Sil1 WT but poorly solubilised in the native cell lysis buffer, we treated the pellet remaining from the native lysis procedure with a strongly denaturing urea buffer (see Section 4). Western blotting analysis of proteins solubilised in urea buffer showed comparable amounts of the three recombinant proteins, confirming that p.E342K and p.L457P are poorly soluble (Figure 3D). These data suggest a selective solubility of Sil1 WT over the variants, with the p.E342K exhibiting a behaviour similar to the pathological p.L457P variant.

Finally, immunofluorescence (IF) microscopy revealed numerous cells containing Sil1 aggregates when COS7 cells were transfected with either the p.E342K or p.L457P variant, whereas only a few cells expressing Sil1 WT showed aggregates (Figure 3E).

Overall, these experiments show that p.E342K behaves like p.L457P and differently from the WT protein, thus indicating that p.E342K is a pathological form of Sil1.

### 2.5. Fibroblasts from Patients Carrying the p.E342K Variant Exhibit a Gene Expression Profile Typical of MSS and Show Reduced Sil1 Levels Due to Active Proteasomal Degradation

To investigate the behaviour of the p.E342K variant in a native environment we generated a primary culture of skin fibroblasts isolated from the patient.

From now on, we will refer to these fibroblasts by the acronym HF-P2, which stands for human fibroblast-patient 2. In the present study, we also used another fibroblast cell line [27], previously obtained from a patient carrying the R111X variant, which will be referred to as HF-P1.

To analyse the functional consequences of p.E342K expression in HF-P2, we monitored the cell cycle, cell growth rate, and cell motility. Cell cycle analysis of the HF-P2 cells revealed a distribution of G1, G2, S, and M phases similar to that of the control fibroblasts (Supplementary Figure S1A,B). Consistently, the proliferation rate of HF-P2 cells was comparable to that of the control cells (Supplementary Figure S1C). In contrast, HF-P1 had a reduced proliferation rate compared to control fibroblasts [27]. Cell motility was also not altered in HF-P2 (Supplementary Figure S1D), whereas it was reduced in HF-P1 [26].

**Figure 4:**
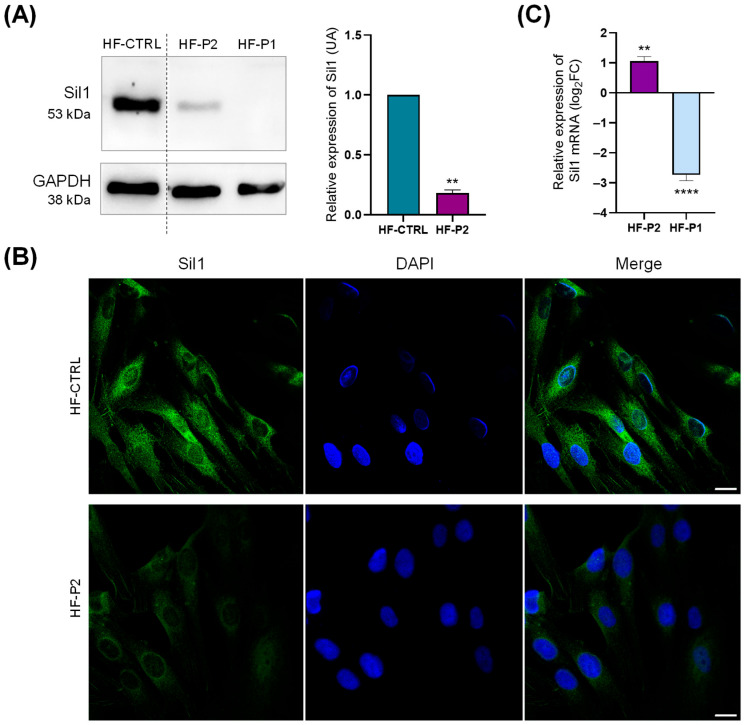
**Sil expression analysis in HF-P2 primary skin fibroblast.** (**A**) Western blotting of control (HF-CTRL), HF-P2, and HF-P1 primary skin fibroblasts. Data are presented as protein expression relative to HF-CTRL. The quantification of Sil1 expression is shown in the graph on the right. GAPDH expression was analysed as loading control. Unpaired t-test was used to compare Sil1 expression of HF-P2 to HF-CTRL. ** *p* < 0.01. (**B**) Confocal IF analysis of Sil1 in HF-P2 cells. The cells were fixed and stained for Sil1 (green), and DAPI (blue) to visualise the nuclei. Merged images are also shown. Scale bar: 20 µm. (**C**) Quantitative RT-qPCR analysis of Sil1 mRNA expression levels in HF-P1 and HF-P2. Results are expressed as the log_2_ of fold changes (log_2_FC) relative to control fibroblasts, calculated using the ΔΔCt method, with GAPDH as the reference gene. Unpaired t-test was used to compare ΔCt of *SIL1* gene between HF-P2 and HF-P1. ** *p* < 0.01 and **** *p* < 0.0001.

Next, we analysed the potential molecular changes occurring in HF-P2. Western blotting and IF analysis showed that Sil1 expression is reduced, by about 80%, in comparison to WT fibroblasts suggesting a protein instability consequent to the amino acid change (Figure 4A,B). Note that Sil1 protein was undetectable in HF-P1 cells carrying a nonsense mutation of Sil1 (Figure 4A). To analyse whether the low expression was due to reduced gene transcription or protein degradation, reverse transcriptase quantitative Polymerase Chain Reaction (RT-qPCR) and Western blot analysis were performed. Sil1 mRNA expression levels, assessed by RT-qPCR, were higher than control healthy fibroblasts (Figure 4C). In contrast, mRNA expression was reduced 5-fold in HF-P1, probably due to the activation of nonsense-mediated mRNA decay (Figure 4C).

**Figure 5:**
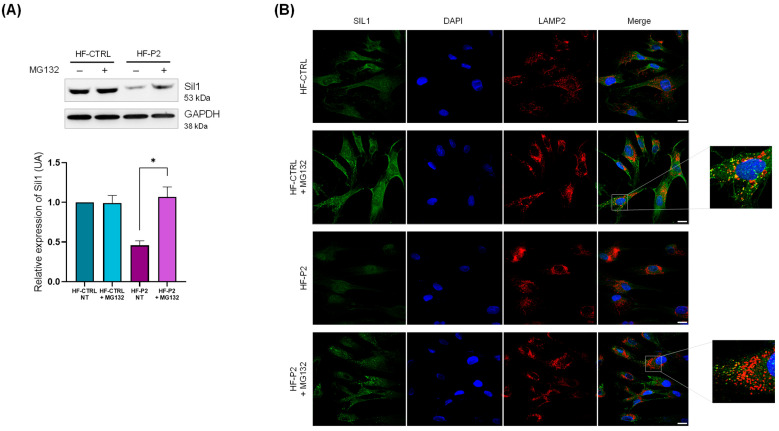
**The Sil p.E342K variant is degraded by the proteasome in HF-P2.** (**A**) Controls and HF-P2 cells were treated with either vehicle or 20 µM MG-132 for 6 h, then lysed and processed for Western blotting. GAPDH expression was analysed as loading control. Quantification of Sil1 expression (from three experiments) of the Western blotting is shown below. Data are presented as protein expression relative to HF-CTRL. Statistical analysis was performed by the paired t-test. * *p* < 0.05. (**B**) Controls and HF-P2 cells were treated as in A. The cells were fixed and stained for Sil1 (green), LAMP2 as lysosomal marker (red) and DAPI (blue) to visualise the nuclei. Merged images are also shown. Scale bar: 20 µm.

To support the hypothesis that the low Sil1 protein expression levels are due to post-translational degradation, we treated HF-P2 with the proteasomal inhibitor carbobenzoxy-L-leucyl-L-leucyl-L-leucinal (MG-132), which indeed significantly increased Sil1 expression (Figure 5A). The same treatment did not significantly affect Sil1 levels in healthy control cells (Figure 5A). The efficacy of MG-132 treatment was confirmed by increased levels of ubiquitinated proteins (Supplementary Figure S2). In addition, IF analysis of cells treated with the proteasome inhibitor showed the formation of numerous Sil1 dots partially co-localised with the lysosomal marker LAMP2 in both control and HF-P2 cells (Figure 5B). These data indicate that lysosomes could be an alternative way for the degradation of unfolded Sil1. We also observed an enlargement of the lysosomal compartment in MG-132-treated cells, which was less evident in HF-P2 cells likely due to the lower levels of Sil1 expression (Figure 5B).

Finally, we evaluated the expression of 12 genes that we previously identified as being altered in HF-P1 [26]. Interestingly, the expression of all these genes was also changed in HF-P2 cells and ten of them moved in the same direction as HF-P1 cells (Supplementary Figure S3). However, it should be noted that the magnitude of gene expression changes observed in HF-P2 was smaller than in HF-P1, suggesting that, although the p.E342K variant is pathological, it might be less severe than R111X.

### 2.6. Fibroblasts from Patients with the p.E342K Variant Exhibit Cytosolic Accumulation of Empty Vesicles, Lipid Droplets, and Fibrotic Material

**Figure 6:**
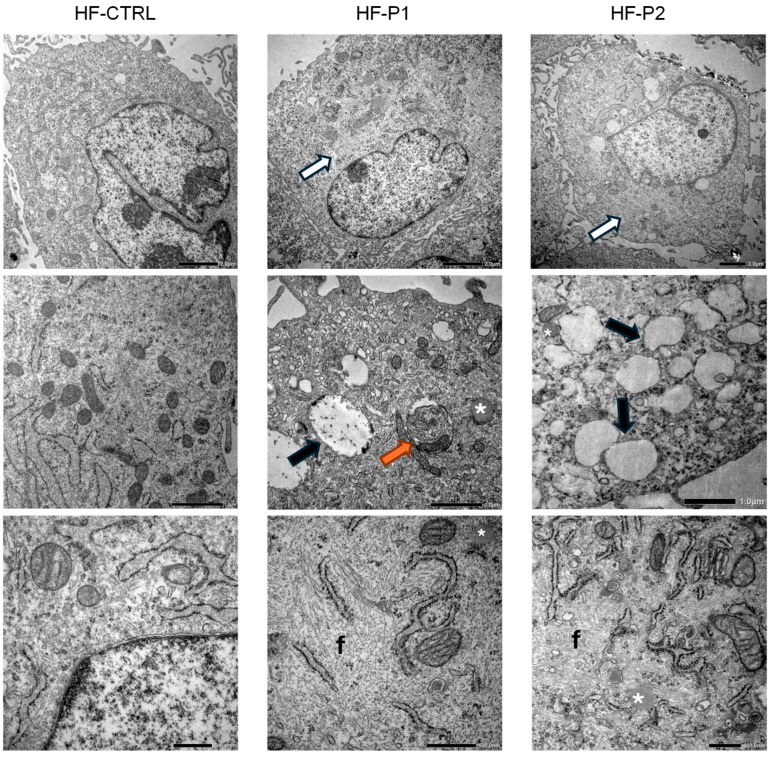
**Ultrastructural analysis of HF-P2 fibroblast.** Transmission electron microscopy (TEM) of control, HF-P1, and HF-P2 cells. HF-P1 and HF-P2 cells exhibited cytoplasmic abnormalities, including clear cytoplasmic material accumulation (white arrows), multilamellar bodies (orange arrows), empty vacuoles (black arrows), lipid droplets (*), and aberrant fibrillar material (f). Scale bars: 2 µm, 1 µm, 500 nm.

To determine if HF-P2 expressing the p.E342K variant exhibited a morphological phenotype, we examined the fibroblasts using transmission electron microscopy (TEM). Ultrastructural examination of HF-P2 cells at low-medium magnification revealed an abundant presence of peripheral variously sized vesicles, which were absent in control cells. Furthermore, unlike in HF-P1 cells, these vesicles appeared predominantly empty (Figure 6, central panels). Cytoplasm of HF-P2 showed depots of lipid droplets (asterisks in Figure 6) and appeared characterised by well-recognisable areas which (i) are clearer than the surrounding cytoplasm (Figure 6, white arrow in upper panels); (ii) are outlined by ER or mitochondria, and (iii) at higher magnification appear to be formed by cytoplasmic deposits/accumulations of proteinaceous fibrillar material (letter f in Figure 6, lower panels). To understand whether this phenotype was specific to HF-P2 or a general characteristic of MSS, we re-analysed HF-P1 by TEM and verified the presence of lipid droplets and fibrotic accumulation in the fibroblasts of both patients (Figure 6). This morphological analysis uncovered new cellular alterations characteristic of MSS and supports the hypothesis that p.E342K is a pathological variant of Sil1.

### 2.7. Proteomic Analysis of Fibroblasts from Patients Carrying the p.E342K Variant Revealed an Altered Proteostasis Characteristic of MSS

**Figure 7:**
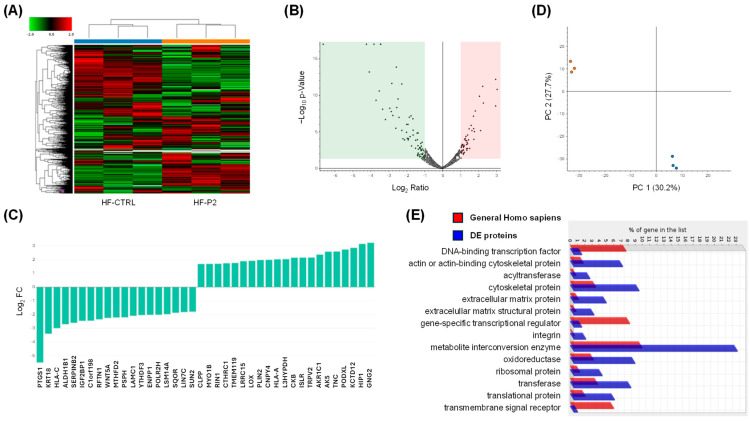
**Comparative proteomic analysis of controls and HF-P2 cells.** (**A**,**B**) Heat map and volcano plot of DE proteins in triplicate experiments. The blue and orange bars in the upper part of the heat map denote the three biological replicates corresponding to HF-CTRL and HF-P2, respectively. Expression ratios and *p*-values are presented on a logarithmic scale in the volcano plot. The green and pink shaded areas highlight proteins with a log_2_ FC greater than 1 or less than −1, respectively, along with −log_10_ (*p*-value) values above 1.3. (**C**) The top 10 upregulated and downregulated DE proteins are expressed as log_2_ FC. (**D**) Principal component (PC) analysis of proteomics data from control (red dots) and HF-P2 cells (blue dots) across three technical replicates. (**E**) Panther protein class identified through ontology enrichment analysis of DE proteins.

To characterise HF-P2 in more detail, we performed a proteomic analysis and identified approximately 2240 proteins; 122 of these were downregulated and 107 were upregulated for a total of 229 differentially expressed (DE) proteins compared to control fibroblasts (*p* < 0.05) (Supplementary Table S1 and Figure 7A).

As a general overview of the proteomic data, the distribution of all proteins based on their log_2_ FC and −log_10_ *p*-value is depicted in the volcano plot (Figure 7B), while the top-modulated proteins, ranked by their FC, are shown in Figure 7C. Principal component analysis of all DE proteins showed that HF-P2 cells cluster distinctly from control cells, further highlighting that the patient cells are markedly different from those of healthy controls (Figure 7D). Enrichment analysis revealed that DE proteins were enriched in cytoskeletal proteins, extracellular matrix proteins and metabolite interconversion enzymes (Figure 7E).

**Figure 8:**
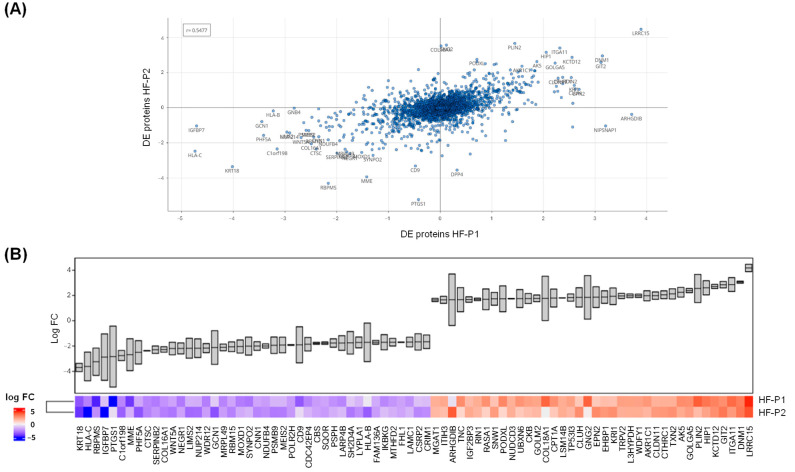
**Comparative analysis of DE proteins identified in HF-P1 and HF-P2 cells.** (**A**) Correlation analysis of the DE proteins identified by proteomics in HF-P1 and HF-P2 cells. (**B**) Comparison of log2 FC in the 40 most upregulated and 40 most downregulated proteins identified by proteomics in HF-P1 and HF-P2 cells.

Interestingly, a comparison of the DE proteins identified in HF-P2 and HF-P1 revealed a general correlation (r = 0.5477) in the alterations observed in both patients’ cells (Figure 8A).

The DE proteins with the most similar level of alteration between patients’ cells are shown in Figure 8B. We would like to emphasise that the proteomics data for HF-P1 cells are not those previously published [27], but were specifically generated for this study. The close correlation observed between HF-P2 and HF-P1 proteomics further supports the hypothesis that p.E342K is a pathological variant.

To further assess the reliability of our proteomics, we validated the expression changes in the proteins: PDZ and LIM domain 5 (PDLIM5), potassium channel tetramerisation domain containing 12 (KCTD12), LIM domain 1 and a half (FHL1), collagen triple helix repeat containing 1 (CTHRC1) and creatine kinase B-type (CKB) by Western blotting analysis. Consistent with the proteomic measurements, altered expression was confirmed for all proteins analysed (Supplementary Figure S4).

### 2.8. Enrichment Analysis of DE Proteins Revealed Alterations in Functional Pathways Previously Reported in MSS

To gain insight into the functional relationship among the DE proteins identified in HF-P2 as well as to infer about the molecular machineries affected by these proteins, we interrogated STRING, a database of protein–protein interaction [40,41]. This analysis showed a protein–protein interaction (PPI) network (Supplementary Figure S5) constituted by 177 nodes (only 52 proteins were not connected) and a significant PPI enrichment (PPI enrichment *p*-value: < 1.0 × 10^−16^) indicative of a biological link among the DE proteins. Functional enrichment analysis according to gene ontology (GO), revealed ribosomal large subunit biogenesis, cytoplasmic translation, regulation of cell adhesion, and oxoacid metabolic process among the biological processes possibly affected in patient cells expressing the p.E342K variant of Sil1. The complete list of biological processes and statistical significance is shown in Supplementary Table S2. Interestingly, a large fraction of these biological processes were the same as those enriched in our previous proteomics (92%) and transcriptomics (53%) of HF-P1 [26,27] as well as in the proteomics (66%) of the *Sil1*^Gt^ mouse quadriceps [14]. Regarding molecular functions, it was revealed that Extracellular matrix structural constituent conferring tensile strength, collagen binding, extracellular matrix structural constituent, mRNA binding and cell adhesion molecule binding were potentially affected. The complete list of molecular functions and statistical significance is shown in Supplementary Table S3. Moreover, among the cellular components enriched in DE proteins there were Cytosolic large ribosomal subunit, Protein complex involved in cell adhesion, collagen trimer, phagocytic vesicle membrane, focal adhesion, endocytic vesicle membrane, collagen-containing extracellular matrix. The complete list of cellular components and statistical significance is shown in Supplementary Table S4. Enrichment of reactome molecular pathways was in line with previous findings on MSS [14,27] and with the above gene ontologies, as it identified insulin-like growth factor-2 mRNA binding proteins (IGF2BPs/IMPs/VICKZs), serine biosynthesis, syndecan interactions, integrin cell surface interactions, collagen chain trimerization, ECM proteoglycans, collagen biosynthesis and modifying enzymes, metabolism of RNA, response of EIF2AK4 (GCN2) to amino acid deficiency, extracellular matrix organisation, metabolism of amino acids and derivatives, and rRNA processing in the nucleus and cytosol. The complete list of pathways and statistical significance is shown in Supplementary Table S5. Similarly, KEGG-enriched pathways confirmed some functional alterations identified in previous studies on MSS (Supplementary Table S6).

In conclusion, proteomic analysis strongly indicates that the p.E342K variant triggers a pathological cellular phenotype very similar to that previously reported for MSS.

## 3. Discussion

MSS is an early-onset neuromuscular disease caused by variants of the *SIL1* gene in about half of the cases, while the gene(s) responsible for the other cases is (are) still unknown [2,7]. We recently examined a young patient with hypotonia and developmental delay; these clinical signs were suggestive of MSS [1]. However, molecular analysis of the *SIL1* gene did not straightforwardly confirm the hypothesised diagnosis. Whole exome sequencing revealed the c.1024G>A variant of unknown significance in the *SIL1* gene. This gene variant generates a mutant protein in which the glutamate at position 342 is replaced by a lysine. The Sil1 protein is a member of the NEF family of heat shock protein 70 kilodaltons (HSP70), which includes 11 members in the human genome. Of these, Sil1 and glucose-regulated protein 170 (Grp170) are localised in the lumen of the ER [19]. It is noteworthy that Grp170 can supplement the loss of Sil1 if overexpressed at high levels, but under pathological conditions, spontaneous overexpression of Grp170 is not sufficient to replace the loss of Sil1 [42]. However, these NEFs, while being functional homologues, are structurally different. Sil1 has an N-terminal signal peptide to direct the newly synthesised protein into the ER lumen, followed by four repeated armadillo domains that are post-translationally modified by a phosphorylation on serine 147, two N-glycosylations at positions 193 and 236, and finally the protein terminates with a putative ER-retention peptide, KELR [10,43,44,45]. The armadillo repeats of Sil1 interacting with the nucleotide-binding domain of BiP promote the release of ADP. Therefore, replacing a negatively charged amino acid with a positively charged one in the middle of the armadillo repeats possibly affects the structure of Sil1 in the area of interaction with BiP and consequently influences their interaction/function. In support of a possible aberrant three-dimensional organisation of the p.E342K variant, we showed that it tends to aggregate and form precipitates. Furthermore, CD spectra indicated a major reduction in the alpha-helical organisation in favour of a coiled-coil fold. The poor solubility of p.E342K variant was confirmed in COS7 cells by Western blotting and IF analysis. This peculiar behaviour has previously been reported for other pathological *SIL1* mutants, including p.L457P, thus supporting the hypothesis that the p.E342K variant is pathological.

To study the p.E342K variant in a native environment and confirm its potential pathogenicity we established a primary skin fibroblast cell line from the patient. Patient-derived fibroblasts are considered an efficient and representative cell model for studying genetic neurodegenerative diseases [46]. We previously reported that skin fibroblasts from an MSS patient exhibited morphological changes and a characteristic gene expression signature consisting of 12 genes [26,27]. In support of the pathological nature of the p.E342K variant, we demonstrated that 10 genes of the MSS signature are affected in the fibroblasts of the patient (HF-P2) under observation. The magnitude of these changes in gene expression was greater in the HF-P1 patient than in the HF-P2 patient, indicating that the new p.E342K variant might be less severe. To date, there is no clear correlation between *SIL1* variants and the severity of the clinical phenotype [6,7].

In HF-P2 fibroblasts, we observed increased Sil1 mRNA levels and a reduction in Sil1 protein, likely due to the misfolding and instability of the mutated protein. The elevated mRNA levels may reflect a compensatory response to the reduced protein expression. Consistent with previous studies, protein degradation depended on a functional proteasome, and its inhibition shunted misfolded proteins into the lysosomal compartment [47]. The importance of the lysosomal compartment in MSS is also underscored by its enlargement, as reported in numerous previous studies including ours [7,16,27]. In the present study, we also reported an accumulation of lipid droplets, a phenotype that could confirm a reorganisation of metabolism in MSS [27]. Indeed, in our study on HF-P1, we reported a reduction in lipid synthesis and an increase in beta-oxidation [27]. Interestingly, several neurodegenerative diseases including amyotrophic lateral sclerosis, Huntington Corea, Parkinson’s, and Alzheimer’s disease pathologies have been linked to the accumulation or dysfunction of lipid droplets [48]. In addition, we reported the presence of fibrotic materials in patient-derived cells a characteristic trait of several neurodegenerative diseases [49].

Proteomic analysis further indicated that the new p.E342K variant is pathological, as the large number of altered proteins in the fibroblast isolated from the patient carrying this variant overlapped those of the HF-P1 patient. At the same time, the biological processes enriched among these DE proteins were in common with those reported to be enriched in the muscle of mouse *Sil1^Gt^*, a model of MSS [14,27]. The comparison of the protein expression profile between HF-P1 and HF-P2 shown in Figure 8A,B underscores this concept.

Finally, we acknowledge several limitations in the present study. First, the findings are based on a single patient, which inherently restricts the generalizability and statistical strength of our conclusions. Second, we did not perform rescue experiments in patient-derived fibroblasts, which would have provided stronger evidence linking the observed phenotype to the p.E342K variant. Third, no in vivo model carrying the p.E342K variant was generated to recapitulate the patient’s phenotype.

## 4. Materials and Methods

### 4.1. Patient Information

The proband is a 4-year-old female who was referred to the Laboratory of Medical Genetics St. Sophia’s Children’s Hospital, Athens, Greece, at 2 years of age for a neurodevelopmental delay. She is the first child of non-consanguineous Greek parents. Her father has two children from previous relationships, a 19-year-old healthy male and a 23-year-old healthy female. The proband also has an 18-year-old maternal half-sister.

### 4.2. Ethical Considerations

Ethical approval for the genetic study was obtained from St. Sophia Children’s Hospital Scientific and Ethics Committee (Approval number: 3669/12-02-18; date of approval: 12 February 2018). All patient data were processed and stored according to the guidelines of the General Data Protection Regulation (GDPR). Written informed consent was provided by the patient’s parents.

### 4.3. Whole-Exome Sequencing and Targeted Sanger Sequencing Analyses

Proband-only WES (~20,000 genes) was performed, using genomic DNA extracted from whole blood. Library preparation was implemented using IDT xGen Exome Research v2 kit (Integrated DNA Technologies, Coralville, IA, USA). The resulting libraries were subjected to paired-end sequencing on an Illumina NextSeq 500 platform (San Diego, CA, USA). WES data from the bioinformatic analysis contained 61,849,178 number of reads and 25,446 variants. The percentage of regions with at least 20× coverage was 98% and the mean coverage was 83×. Variant analysis was performed using VarSome Clinical platform (Saphetor SA, Lausanne, Switzerland) [50,51] and was based on the phenotype-driven strategy [52]. Variant classification was based on ACMG guidelines [53].

Targeted Sanger sequencing for the detected variant was performed in the proband and her parents. PCR for amplification of exon 9 of the *SIL1* gene was performed using specific primers designed with NCBI Primer Blast [54,55]. The primer sequences flanking exon 9 of the *SIL1* were 5′-GGGTATAGCTGTAACTGAGGTG-3′ (forward) and 5′-CTCACATGACATACTCCCTGAC-3′ (reverse). PCRs were performed using Hot Start Taq (Qiagen, Hilden, Germany) polymerase and both strands were sequenced using the Big Dye^®^ Terminator v3.1 Cycle Sequencing Kit (Thermo Fisher Scientific SA, Waltham, MA, USA). Capillary electrophoresis was performed in ABI 3500 Genetic Analyzer sequencing system (Thermo Fisher Scientific SA, Waltham, MA, USA).

### 4.4. Preparation of Recombinant Sil1

To express Sil1 in COS7 cells, the ORF of the human *SIL1* gene, its variant c.1024G>A (p.E342K) and the pathogenic missense variant c.1370T>C (p.L457P) were cloned into the mammalian gene expression vector pRP-CMV (VectorBuilder Inc., Chicago, IL, USA). 

To express Sil1 in *Escherichia coli* (*E. coli*), the *SIL1* WT and *SIL1* p.E432K variant, both lacking the first 31 amino acids at the N-terminus, were cloned into the pGEX6P1 vector (GenScript, Piscataway, NJ, USA), enabling the recombinant proteins to have an N-terminal GST tag. BL21(DE3) competent cells (Novagen, Houston, TX, USA) were transformed with 100 ng of plasmid, and colonies were grown at 37 °C on Luria–Bertani (LB) agar plates containing ampicillin 50 mg/mL. Transformed bacteria were suspended in LB Miller broth until the absorbance at 600 nm reached 0.5–0.6. Expression of the recombinant proteins was induced by 0.5 mM isopropyl-β-D-thiogalactopyranoside for 3 h at 25 °C. After centrifugation at 4000× *g* for 15 min at 4 °C, the bacterial pellets were frozen at −80 °C until use. The pellets were resuspended in HEPES 50 mM, NaCl 400 mM, pH 7.4 and 10% glycerol, DNAse I and a cocktail of protease inhibitors and lysed for 30 min by sonication on ice (10 s on, 10 s off; amplitude 60% cycle 0.5). Lysates were then centrifuged at 16,000× *g* for 1 h at 4 °C, and the supernatant was loaded onto Glutathione Sepharose 4B agarose resin, equilibrated in HEPES 50 mM, NaCl 400 mM pH 7.4. The affinity chromatography purification was carried out at room temperature. The column was first washed in HEPES 50 mM, NaCl 500 mM pH 7.4 with the addition of ATP 5 mM and MgCl_2_ 15 mM to remove potential contamination from bacterial chaperones. The resin was incubated overnight with human rhinovirus 14 3C protease to cleave the GST-tag and the recombinant Sil1 WT, Sil1 p.E432K were eluted in HEPES 50 mM, NaCl 400 mM pH 7.4. All the eluted fractions were analysed by 10% SDS-PAGE under reducing conditions and stained overnight with ReadyBlue Protein Gel Stain (Sigma-Aldrich, St. Louis, MO, USA).

The purified proteins were then concentrated with Amicon devices (MWCO 10 kDa, Sigma-Aldrich, St. Louis, MO, USA) and the protein concentration was estimated by measuring the absorbance at 280 nm using ε = 33.015 deduced by ProtParam [56].

### 4.5. CD Analysis of the Recombinant Sil1

Sil1 WT and Sil1 p.E432K proteins were dialysed against Tris/acetate 20 mM, NaF 200 mM pH 7.4 and analysed in Chirascan system (Applied Photophysics, SFR BioSciences, Lyon, France). CD spectra were recorded at 15 °C in a quartz cuvette with a thickness of 1 mm in the range of 180 nm to 350 nm with a step size of 0.5 nm and a bandwidth of 1 nm. Four spectra were collected and averaged for each sample, with background and buffer signals subtracted. Sil1 p.E432K spectra were also recorded in presence of 10% of 2,2,2-trifluoroethanol (Sigma-Aldrich, St. Louis, MO, USA).

### 4.6. Cell Culture

Cells were cultivated at 37 °C in a CO_2_ incubator, using Dulbecco’s modified Eagle’s medium GLUTAMAX (catalogue number 61965-026) supplemented with 10% of Fetal Bovine Serum (GIBCO) and 1% penicillin/streptomycin (catalogue number 15070-063). Cells were detached with 0.5% Trypsin/EDTA (catalogue number 15400-054) and subcultured at 90% of confluence.

In this study, four cell lines were used: COS-7 cells, available in our laboratory [57,58]; HF-P1 primary human dermal fibroblasts derived from a young patient with Marinesco–Sjögren syndrome, provided by the Telethon Network of Genetic Biobanks (TNGB) and characterised in our previous study [27,59]; HF-P2 primary skin fibroblasts were established from a punch biopsy of the patient carrying the VUS. These cells were grown in minimal essential medium with Earl salts supplemented with 15% (*v*/*v*) non-inactivated Fetal Bovine Serum (ByProductos, Guadalajara, Jalisco, Mexico), 1% Glutamax, 1% penicillin/streptomycin, and 1% non-essential amino acids (In vitro, Mexico City, Mexico). Cells were maintained in an incubator at 37 °C and 5% CO_2_/95% atmospheric air. The culture medium was changed every third day until cells reached 90% of confluence. Once the cell line was established, HF-P2 cells were cultured following the same procedures described above for the other cell lines used in this study. Finally, primary human dermal fibroblasts from a healthy donor were used as controls (NDHF, PromoCell, catalogue number FB60C12350; Carlo Erba Reagents, Cornaredo, Italy). Unless otherwise specified, all cell culture reagents were obtained from Gibco (Life Technologies, Grand Island, NY, USA).

### 4.7. Culturing and Characterisation of Primary Skin Fibroblasts

#### 4.7.1. Cell Cycle Analysis

Cell cycle phases were determined using Propidium Iodide (#P1304MP, Thermo Fisher Scientific, Waltham, MA, USA). Control and HF-P2 were detached from Petri dishes and resuspended at a concentration of 5 × 10^5^ cells/well. The cells were washed with PBS, fixed in 70% cold ethanol for 3 h at 4 °C, and then incubated with PI solution (PBS, PI, RNase A #A797C Promega, Madison, WI, USA), in the dark at +4 °C ON. Next, 10,000 events/sec were acquired with BD FACS Canto II flow cytometry (BD Bioscience, Franklin Lakes, NJ, USA). The data were analysed using FLOWJO software v10.10 (BD Bioscience, Franklin Lakes, NJ, USA). Statistical analyses were performed with GraphPad Prism 5.0 (GraphPad Software, Inc., San Diego, CA, USA).

#### 4.7.2. Cell Proliferation Assay

Cells were plated in a 96-well plate at a concentration of 10,000 cells per well the day before the experiment. Proliferation was then monitored using the Click-iT Edu Proliferation Assay Kit (Thermo Fisher Scientific, Waltham, MA, USA; catalogue number #C10499) according to the manufacturer’s instructions. Unpaired t-test was performed to compare the proliferation rate.

#### 4.7.3. Motility Assay

Following the Incucyte Scratch Wound Assay protocol, 20,000 cells/well were placed in a 96-well ImageLock™ plate the day before the experiment. The day after, a uniform wound was created in each well through the 96-pin Incucyte WoundMaker (Sartorius, Göttingen, Germany), and then cells were incubated into Incucyte^®^ Live Cell Analysis System (Incucyte^®^ S3/-Sartorius). Image acquisition and analysis of relative wound density were performed following our previously published protocol [26].

#### 4.7.4. Western Blot Analysis

Controls and patient-derived fibroblasts were grown to 70% of confluency, lysed and processed for Western blotting as previously described [26,27]. Primary antibodies used for protein detection are listed in Supplementary Table S7.

### 4.8. COS7 Cell Transfection, Treatments, and Western Blot Analysis

For transfection, 250,000 COS7 cells/well were plated in a 6-well plate containing a coverslip in each well. The following day, the cells were transfected with 3 µg of, respectively, *SIL1* WT, *SIL1* p.E342K, and *SIL1* p.L457P, using Lipofectamine 3000 (Thermo Fisher Scientific, Waltham, MA, USA, catalogue number L3000015) according to manufacturer’s instructions. For IF analysis, after 18 h of transfection, the cells were treated for 2 h with cycloheximide (25 µg/mL) and fixed. For Western blot analysis, transfection was terminated after 72 h, and cells were lysed and processed as described in our previously published studies [26,27,60] with the exception of the use of precast gel (Genscript, Piscataway, NJ, USA, catalogue number M00659 SurePAGE™, Bis-Tris, 10 cm × 8 cm, 8–16%, 12 wells). MG132 proteasome inhibition was performed by seeding the cells at 70% of confluency in 60 mm or in 24-well dishes containing coverslips. The day after, the cells were treated for 6 h with MG132 20µM (Calbiochem, San Diego, CA, USA, catalog number 474790). Finally, the cells have been processed for Western blotting and IF confocal microscopy as appropriate. 

### 4.9. Fluorescence Immunostaining and Confocal Microscopy 

Cells seeded on coverslips were fixed in ice-cold methanol at −20 °C for 5 min once they reached 70% confluence. Cells were then processed according to our previously published studies [27,60]. Primary antibodies used in IF are listed in Supplementary Table S7. Fluorescence was detected using an LSM800 Zeiss confocal microscope (Carl Zeiss, Jena, Germany) and images were post-processed and analysed using Fiji/ImageJ version 2.16.0/1.54p (National Institutes of Health, Bethesda, MD, USA).

### 4.10. Native-PAGE

Cells were lysed under gentle conditions as described by Roychowdhury et al. [61]. The native lysis buffer consisted of Tris 20 mM (pH 7.4), KCl 20 mM, MgCl_2_ 5 mM, and 0.01% NP40, supplemented with protease and phosphatase inhibitor cocktails (Merck, Darmstadt, Germany). Cells suspended in the lysis buffer were incubated for 10 min on dry ice, followed by 2 min at 37 °C in a water bath. This step was repeated three times, then the samples were incubated on ice for 30 min. After centrifugation for 20 min at 12,000 rpm at 4 °C, both the supernatants and pellets were collected. Pellets were treated on ice for 30 min at 4 °C with native lysis buffer added with Urea 8 M, then sonicated and centrifuged for 20 min at 4 °C (12,000 rpm). Protein concentrations were measured with the Bradford assay. 

Proteins solubilised in native buffers were analysed by native-PAGE, while proteins solubilised in urea buffer were analysed by SDS-PAGE. For native-PAGE electrophoresis, stacking gel (4% acrylamide) running gel (10% acrylamide) and running buffer (1×) were prepared without SDS, according to the indications of Sino Biological Inc [62]. Proteins loaded on native-PAGE were not heated but simply diluted with Tris-HCl 62.5 mM pH 6.8, 25% glycerol and 1% Bromophenol Blue. The electrophoresis run was performed at 100 V with the Bio-Rad apparatus placed on ice (Bio-Rad Laboratories, Hercules, CA, USA).

### 4.11. RNA Extraction and RT-qPCR

Total RNA was extracted from cells using QIAzol (RNeasy Lipid Kit, Qiagen, Hilden, Germany), according to the manufacturer’s instructions. RNA quality and concentration were determined by NanoDrop 2000 spectrophotometry (Thermo Fisher Scientific, Waltham, MA, USA) and verified by 1% agarose gel electrophoresis. A total of 1 µg of total RNA was reverse transcribed into cDNA using the High Capacity cDNA Archive kit in accordance with the manufacturer’s recommendations (Thermo Fisher Scientific, Waltham, MA, USA).

Relative quantification of gene expression was performed by RT-qPCR using the CFX96 Real-Time PCR Detection System (Bio-Rad, Laboratories Hercules, CA, USA) and the SensiFAST SYBR kit (Bioline, Meridian Bioscience, Cincinnati, OH, USA). Primers used for amplification were designed using IDT PrimerQuest Tool (Primer3 software version 2.2.3) and synthesised by Integrated DNA Technologies (IDT, Coralville, IA, USA). The primer sequences are shown in Supplementary Table S8. Data analysis was performed using CFX Manager software version 3.1 (Bio-Rad Laboratories, Hercules, CA, USA). The relative expression of target genes was calculated using the ΔΔCt method, normalising the expression of target genes to the reference gene (GAPDH) [63]. The results are shown as log_2_ FC with respect to the control cells. Multiple unpaired t-test was used to compare ΔCt of *SIL1* gene between HF-P2 and HF-CTRL.

### 4.12. TEM Analysis

Approximately 2 × 10^6^ fibroblast cells cultured in monolayers were prepared for TEM as previously described [27,60]. Cells were detached from the dish with trypsin, centrifuged at 180× *g* for 2.5 min, washed 3 times with phosphate buffered saline (PBS) at 37 °C, fixed with 3.5% glutaraldehyde in sodium cacodylate buffer (NaCaCO) 0.1 M for 1 h, and stored at 4 °C. For embedding, cells were post-fixed in 2% OsO4 in the same buffer for 2 h and block-stained in saturated uranyl acetate replacement. After dehydration, specimens were embedded in epoxy resin (Epon 812, Electron Microscopy Sciences, Hatfield, PA, USA). Ultrathin sections were cut using a Leica Ultracut R microtome (Leica Microsystem, Wien, Austria) with a Diatome knife (Diatome Ltd. CH-2501, Biel, Switzerland) and double-stained with uranyl acetate replacement and lead citrate. Sections were viewed and photographed in a JEM-1400 Flash transmission electron microscope (Jeol Ltd., Tokyo, Japan) equipped with a Matataki digital camera and SightX Viewer software (ver.2.1.26.1818, Jeol Ltd., Tokyo, Japan).

### 4.13. Label-Free Proteomics and DE Protein Data Analysis

Cell samples were lysed in buffer B Strong (UREA 6 M, Tris Base 100 mM, CHAPS 2%, Triton X 1%, DTT 50 mM), and treated by Filter Aided Sample Preparation method. As described previously [27] protein concentration was evaluated through Bradford assay (Bio-Rad Laboratories, Hercules, CA, USA) to digest 30 µg of protein by trypsin (0.5 µg/µL Trypsin, Sigma-Aldrich, St. Louis, MO, USA). Tryptic peptides were analysed in triplicate with nano-liquid chromatography-mass spectrometry (LC-MS/MS) using the UltiMate^TM^ 3000 UPLC (Thermo Fisher Scientific, Waltham, MA, USA) chromatographic system coupled to the Orbitrap Fusion^TM^ Tribrid^TM^ (Thermo Fisher Scientific, Waltham, MA, USA) mass spectrometer with the EASY-spray Acclaim^TM^ PepMap^TM^ C18 (75 μm ID, 15 cm L, 2 μm PS, Thermo Fisher Scientific) chromatographic column in a total run time of 65 min and a chromatographic gradient from 5% to 90% of acetonitrile. Label-free protein identification and quantification were as in Di Stefano et al. [64]. Raw data were processed using Thermo Proteome Discoverer (PD) version 2.4.0.305 (Thermo Fisher Scientific, Waltham, MA, USA). In order to perform a robust quantification, we removed all contaminant proteins from the dataset and set the parameters “Abundance Ratio Variabilities [%]” less than 50.00 in the ratio and number of “Unique peptides” greater than one. The volcano plot was generated from the statistical analysis performed using PD software version 2.4 and highlights significantly up- and down-regulated proteins, with the *p*-value indicating the statistical significance of the “Protein Abundance Ratio”. Similarly, the heatmap was generated using PD software, considering the individual protein abundance values for each quantified protein in each analytical replicate. Raw protein abundances were loaded and analysed using BigOmics Analytics: Omics Analysis Software version 3.5 (BigOmics Analytics, Lugano, Switzerland) [65].

## 5. Conclusions

The present study reports a previously undescribed c.1024G>A variant in the *SIL1* gene, which encodes a key protein involved in protein folding within the ER. The alteration of the *SIL1* gene is linked to the development of MSS. In-depth analyses, including in silico predictions, circular dichroism, and native gel electrophoresis, suggest that the novel p.E342K variant disrupts the structure and function of the Sil1 protein, leading to its degradation. This issue leads to a significant reduction in Sil1 protein levels in the patient’s fibroblasts, resulting from proteasomal degradation. The patient’s fibroblasts exhibited a proteomic expression profile similar to a known MSS case and mouse model, along with a distinct transcriptional signature characteristic of the disease. Additionally, ultrastructural analysis revealed autophagic vacuoles and a previously unreported accumulation of lipid droplets, findings consistent with those observed in other MSS patient fibroblasts. These observations support the reclassification of the new SIL1 c.1024G>A (p.E342K) variant as pathogenic following the ACMG/AMP 2015 guidelines and refined by ClinGen SVI.

By illustrating how this variant results in impaired Sil1 protein function, the study links a clear molecular defect to clinical symptoms, providing valuable insights for diagnosis. In fact, this research provides evidence regarding the genotype–phenotype relationship in MSS, as the phenotype associated with the novel p.E342K variant appeared less severe than that caused by the R111X missense variant. Finally, the study’s findings inform future genetic testing and counselling for MSS patients.

## Data Availability

The original contributions presented in this study are included in the article and the Supplementary Materials. Further inquiries can be directed to the corresponding author. The mass spectrometry proteomics data have been deposited to the ProteomeXchange Consortium via the PRIDE [66] partner repository with the dataset identifier PXD062620.

## Supplementary Materials

The supporting information can be downloaded at: https://www.mdpi.com/article/10.3390/ijms262311310/s1.

## Abbreviations

The following abbreviations are used in this manuscript:

ACMG	American College of Medical Genetics
Arg	Arginine
ATP	Adenosine triphosphate
BiP	Binding immunoglobulin protein
BS3	Benign Strong
CD	Circular dichroism
CKB	Creatine kinase B-type
ClinGen	Clinical Genome Resource
CTHRC1	Collagen triple helix repeat containing 1
DAPI	4′,6-Diamidino-2-phenylindole dihydrochloride
DE	Differentially expressed
ER	Endoplasmic reticulum
FC	Fold change
FHL1	LIM domain 1 and a half
GAPDH	Glyceraldehyde-3-phosphate dehydrogenase
GDPR	General Data Protection Regulation
Glu	Glutamic acid
Gln	Glutamine
GO	Gene ontology
Grp170	Glucose-regulated protein 170
GST	Glutathione S-transferase
His	Histidine
HSP70	Heat shock protein 70 kilodaltons
IF	Immunofluorescence
INPP5K	Inositol polyphosphate-5-phosphatase K
KCTD12	Potassium channel tetramerization domain containing 12
LAMP2	Lysosome-associated membrane protein 2
LB	Luria–Bertani
LC-MS/MS	Liquid chromatography-mass spectrometry
Lys	Lysine
MG-132	Carbobenzoxy-L-leucyl-L-leucyl-L-leucinal
MSS	Marinesco-Sjögren syndrome
NEF	Nucleotide exchange factor
PAGE	Polyacrylamide gel electrophoresis
PBS	Phosphate buffered saline
PD	Proteome Discoverer
PDLIM5	PDZ and LIM domain 5
PPI	Protein–protein interaction
PS3	Pathogenic Strong
RT-qPCR	Reverse transcriptase quantitative polymerase chain reaction
SDS	Sodium dodecyl sulphate
SVI	Sequence Variant Interpretation
TEM	Transmission electron microscopy
TFE	Trifluoroethanol
UPR	Unfolded protein responses
VUS	Variant of unknown significance
WES	Whole-exome sequencing
WT	Wild type
