# Supplementary material for: A Novel SIL1 Variant (p.E342K) Associated with Marinesco–Sjögren Syndrome Impairs Protein Stability and Function
Source: Int J Mol Sci. 2025 Nov 22;26(23):11310. doi: 10.3390/ijms262311310 (PMC12691736; doi:10.3390/ijms262311310)
Supplement: Supplementary file 1 [file ijms-26-11310-s001.zip › ijms-3935520-supplementary/Supplementary Figures.pdf]

Supplementary Figures

Article

A Novel *SIL1* Variant (p.E342K) Associated with Marinesco-Sjögren Syndrome Impairs Protein Stability and Function

Anna Giulia Ruggieri [1,2,†], Nikolaos M. Marinakis [3,4,†], Laura Amodei [1,2], Francesca Potenza [1,2], Afrodite Kampouraki [3], Faidon-Nikolaos Tilemis [3], Laura Pietrangelo [5], Marianna Viele [1,2], Federica Di Marco [1,2], Piero Del Boccio [2,6], Federica Di Cintio [2,7], Nikoletta Selenti [3], Manthoula Valari [8], Luca Federici [1,2], Adriana Erica Miele [9,10], Michele Sallese [1,2,*,‡] and Periklis Makrythanasis [3,‡]

[1] Department of Innovative Technologies in Medicine and Dentistry, "G. d'Annunzio" University of Chieti-Pescara, 66100 Chieti, Italy; annagiulia.ruggieri@unich.it (A.G.R.); laura.amodei@unich.it (L.A.); francesca.potenza@unich.it (F.P.); marianna.viele@phd.unich.it (M.V.); federica.dimarco@unich.it (F.D.M.); luca.federici@unich.it (L.F.)
[2] Center for Advanced Studies and Technology (CAST), "G. d'Annunzio" University of Chieti-Pescara, 66100 Chieti, Italy; p.delboccio@unich.it (P.D.B.); federica.dicintio@unich.it (F.D.C.)
[3] Laboratory of Medical Genetics, Medical School, St. Sophia's Children's Hospital, National and Kapodistrian University of Athens, 115 27 Athens, Greece; nikomari@med.uoa.gr (N.M.M.); afkampouraki@med.uoa.gr (A.K.); ftilemis@med.uoa.gr (F.-N.T.); nselenti@med.uoa.gr (N.S.); pmakryth@med.uoa.gr (P.M.)
[4] Laboratory of Genetics, Department of Medicine, Democritus University of Thrace, 68100 Alexandroupolis, Greece
[5] Department of Medicine and Aging Sciences,"G. d'Annunzio" University of Chieti-Pescara, 66100 Chieti, Italy; laura.pietrangelo@unich.it
[6] Department of Sciences, "G. d'Annunzio" University of Chieti-Pescara, 66100 Chieti, Italy
[7] Department of Medical, Oral and Biotechnological Sciences, "G. d'Annunzio" University of Chieti-Pescara, 66100 Chieti, Italy
[8] Department of Dermatology, Medical School, St. Sophia's Children's Hospital, National and Kapodistrian University of Athens, 115 27 Athens, Greece; valarimd@gmail.com
[9] Department of Biochemical Sciences, Sapienza University of Rome, P. Le Aldo Moro 5, 00185 Rome, Italy; adriana.miele@univ-lyon1.fr
[10] Institute of Analytical Sciences, UMR 5280 ISA CNRS UCBL, Université Claude Bernard Lyon 1, 5 Rue de la Doua, 69100 Villeurbanne, France
* Correspondence: michele.sallese@unich.it; Tel.: +39-0871-541427
† These authors contributed equally as first authors.
‡ These authors contributed equally as last authors.

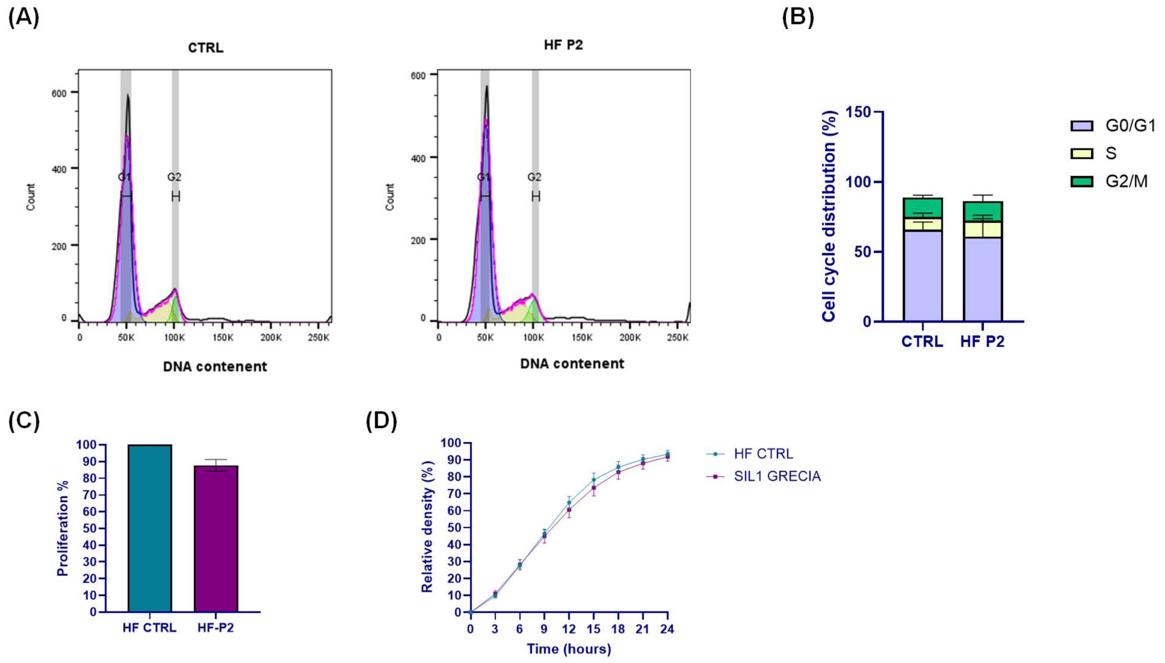

Supplementary Figure S1: Proliferation and motility assays assessing HF-P2 cell behaviour. A) Flow cytometry analysis of the cell cycle of control (CTRL) and HF-P2 cells. B) Percentage of cells analysed in panel A across the different cell cycle phases. C) Thymidine analogue (EdU)-based proliferation assay of control (CTRL) and HF-P2 cells. Data are presented as a percentage relative to the control cells. . D) Wound-healing motility assay of control (CTRL) and HF-P2 cells conducted using the Incucyte system.

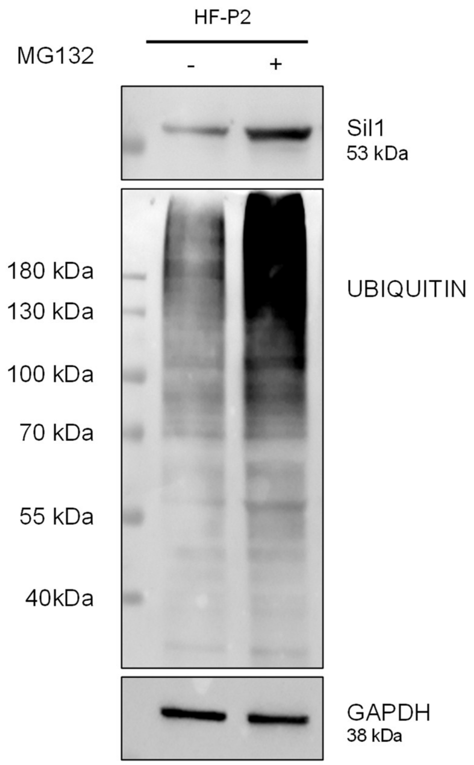

Supplementary Figure S2. Ubiquitination analysis of HF-P2 cells treated with MG-132. HF-P2 cells were exposed to MG-132 20 μM for 6 hours, then lysed and processed for Western blotting using anti-ubiquitin and anti-Sil1 antibodies as indicated. GAPDH expression was analysed as loading control.

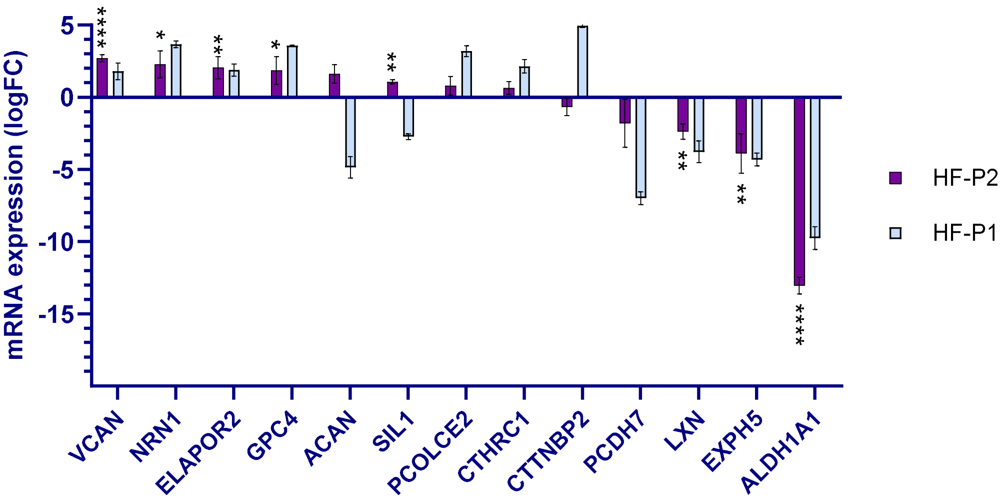

Supplementary Figure S3. **RT-qPCR analysis of selected genes.** Total RNA was extracted from controls, HF-P1 and HF-P2 cells and gene expression analysed by qPCR. Data are expressed as the log of fold change relative to control, calculated using the ΔΔCt method, with GAPDH as the reference gene. Multiple unpaired t-test was used to compare ΔCt of *SIL1* gene between HF-P2 and HF-CTRL. * p <0.05, ** p <0.01, *** p <0.001, **** p <0.0001.

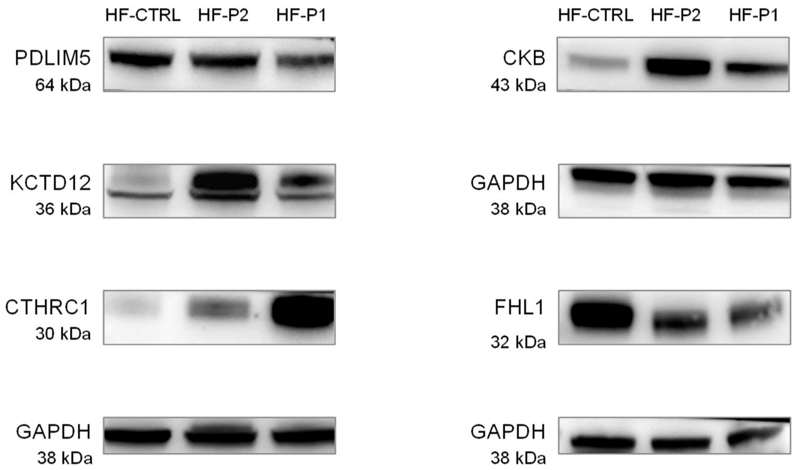

Supplementary Figure S4. Validation of selected DE proteins by Western blot. Control (CTRL) and HF-P2 cells were subjected to Western blot analysis, to analyse the expression of PDLIM5, KCTD12, CTHRC1, CKB, FHL1 and Sil1 as indicated. GAPDH expression was analysed as loading control.

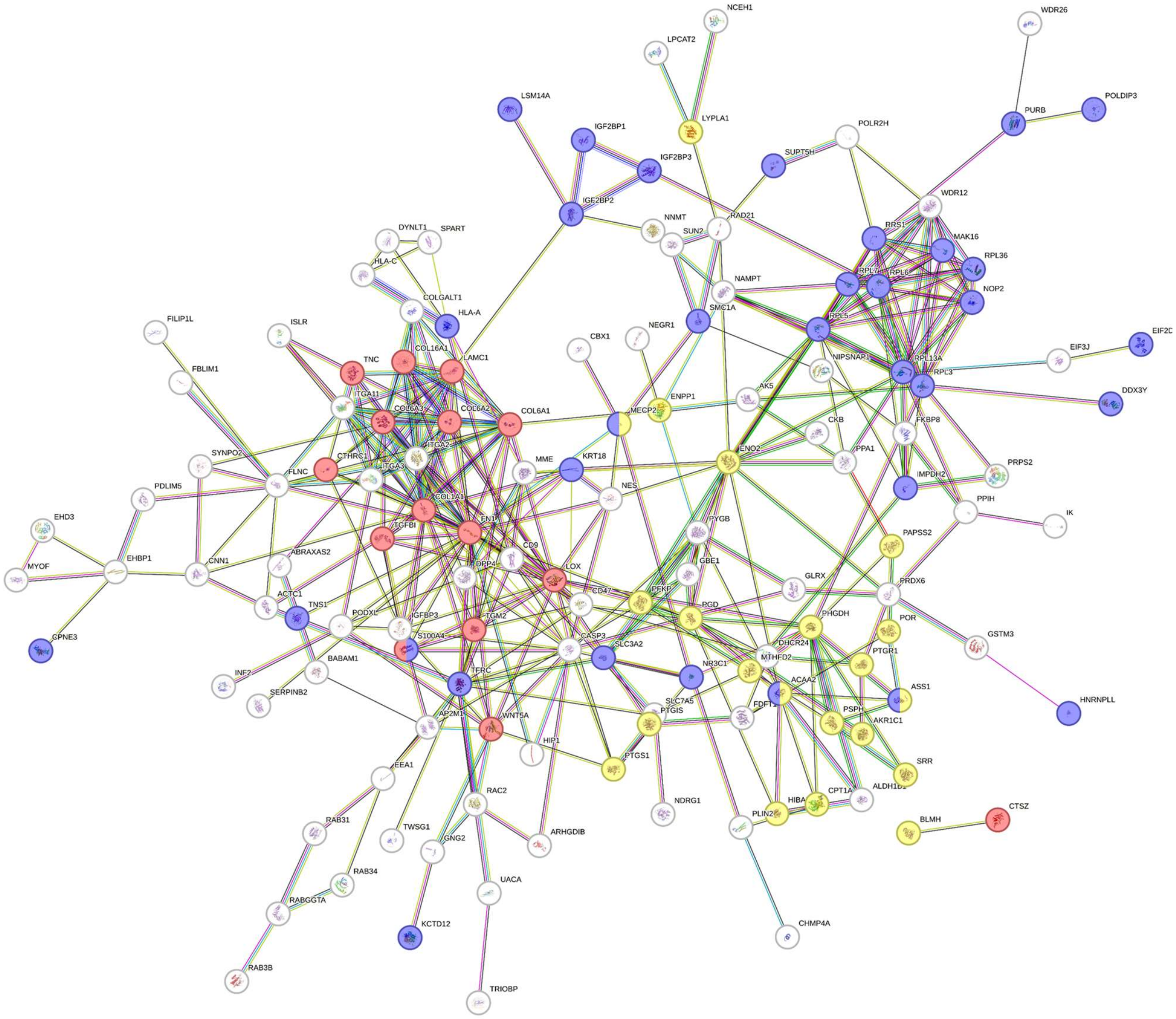

Supplementary Figure S5. Protein-protein interaction network of DE proteins identified in HF-P2 cells. Proteins associated with the biological process 'Oxoacid metabolic process,' the molecular function 'RNA binding,' and the cellular component 'Collagen-containing extracellular matrix' are marked in yellow, blue, and red, respectively.
